# Variants in the vitamin D receptor gene and asthma

**DOI:** 10.1186/1471-2156-6-2

**Published:** 2005-01-15

**Authors:** Matthias Wjst

**Affiliations:** 1 Gruppe Molekulare Epidemiologie, Institut für Epidemiologie, GSF – Forschungszentrum für Umwelt und Gesundheit, Ingolstädter Landstrasse 1, D-85758 Neuherberg / Munich, Germany

## Abstract

**Background:**

Early lifetime exposure to dietary or supplementary vitamin D has been predicted to be a risk factor for later allergy. Twin studies suggest that response to vitamin D exposure might be influenced by genetic factors. As these effects are primarily mediated through the vitamin D receptor (VDR), single base variants in this gene may be risk factors for asthma or allergy.

**Results:**

951 individuals from 224 pedigrees with at least 2 asthmatic children were analyzed for 13 SNPs in the VDR. There was no preferential transmission to children with asthma. In their unaffected sibs, however, one allele in the 5' region was 0.5-fold undertransmitted (p = 0.049), while two other alleles in the 3' terminal region were 2-fold over-transmitted (p = 0.013 and 0.018). An association was also seen with bronchial hyperreactivity against methacholine and with specific immunoglobulin E serum levels.

**Conclusion:**

The transmission disequilibrium in unaffected sibs of otherwise multiple-affected families seem to be a powerful statistical test. A preferential transmission of vitamin D receptor variants to children with asthma could not be confirmed but raises the possibility of a protective effect for unaffected children.

## Background

Early exposure to dietary and supplementary vitamin D has been predicted to be a risk factor for later allergy and asthma [1]. Supported by *in vitro *[2] and *in vivo *studies [3], also epidemiological studies [4,5] report a positive association between supplementary vitamin D use and later allergy [6].

Vitamin D has been used for many years in various doses and preparations to prevent rickets, a disease usually induced by poor dietary calcium intake and sun deprivation. It seems that widespread "historical" rickets in industrial countries was also a genetic disease. A formal twin analysis yielded a 91% concordance rate in monozygotic twins compared to 23% in dizygotic twins [7]. Also a very recent study of baseline gene expression in lymphoblastoid cell-lines found the expression of at least four vitamin D related genes as a heritable trait [pers. comm. Monks 2004], which also makes a genetically determined vitamin D sensitivity likely. It may be speculated that common rickets is the low sensitivity form (in the absence of proper endogenous vitamin D production), and allergy the high sensitivity form (in the presence of high oral vitamin D exposure).

**Figure 1:**
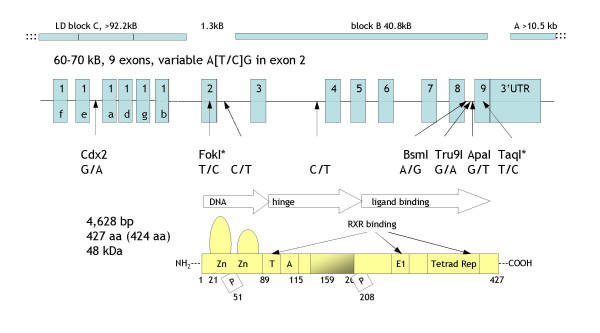
Structure of the vitamin D receptor [40, 41]. Upper: LD blocks in Caucasians [14] where blocks "C" and "A" extend to both sides. In Africans block "C" is split into 3 parts, "C1", "C2" and "C3". Middle: Exon structure including several SNP variants examined in about 100 disease-association studies. Lower: Aligned protein domains, DNA binding, hinge and ligand binding region including phosphorylation sites.

The active vitamin D metabolite 1,25(OH)_2_D_3 _binds to nuclear vitamin D receptor (VDR), which exists from under 500 to over 25,000 copies / cell in many human tissues including thymus, bone marrow, B and T cells and lung alveolar cells [8]. The gene for VDR was cloned in 1988, it consists of 9 exons with at least 6 isoforms of exon 1 and spans 60–70 kb of genomic sequence (Fig. 1) [8]. The VDR is also a first-order positional candidate as nearly all asthma and allergy linkage studies found linkage on chromosome 12q [9,10]. While an own study of a single *Fok1 *restriction site (that alters the ATG start codon in the second exon of the VDR) in asthma families did not find an association [11], positive association of several VDR variants with asthma has been shown in the meantime by two U.S. [12] as well as one Canadian study [13].

So far, dbSNP catalogued 117 SNPs in the VDR when a resequencing approach of the VDR published in June 2004 found 245 SNPs [14]. In this study three LD blocks were localized. Block "A" at the 3' end of exon 9 spans approximately 10.5 kb. VDR exons 3 – 9 are situated in block "B", which spans 40.8 kb. A 5.7 kb LD-breaking spot separates blocks "A" and "B", while blocks "B" and "C" are separated by a 1.3 kb LD-breaking spot; this region also includes VDR exon 2 and the commonly studied *FokI *SNP. All three LD blocks have now been covered by additional SNPs in our asthma family sample.

## Results

The sample analyzed here consisted of 951 individuals from 224 pedigrees contributing 11,383 genotypes.

Mean pedigree size was 4.4; the number of phase-known individuals ranged from 221 to 305 (with the exception of rs2853563) in affected, and from 24 to 41 in the unaffected, children. Except for rs2853563, the minor allele frequency always exceeded 19%. In two families both parents had asthma, in 82 only one parent had asthma and in 140 families both parents were disease-free.

**Figure 2:**
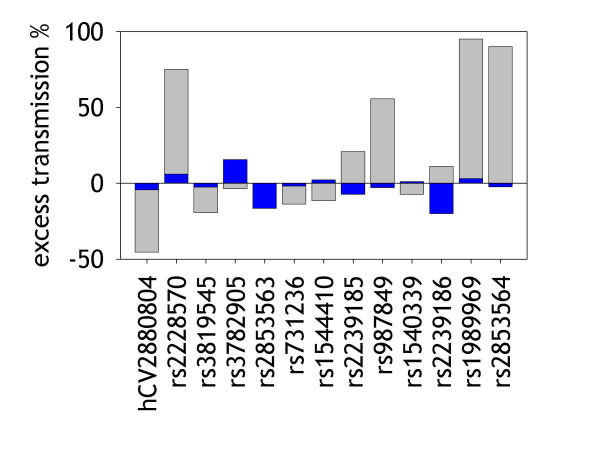
Excess transmission of VDR-SNPs in asthma families. Blue bars indicate transmission to affected children, grey bars transmission to unaffected children.

Of the markers tested, only marker rs2239186 showed a slightly reduced transmission ratio in asthmatic children (0.8, P = 0.073). In unaffected children three markers showed significantly altered ratios: 0.5 for hCV2880804, and 2.0 and 1.9 in rs1989969 and rs2853564, respectively. Excess transmission was generally more pronounced in unaffected children (Fig. 2), however, the three significant associations would also not persist if adjusted for multiple testing with the method described by Bonferroni.

**Figure 3:**
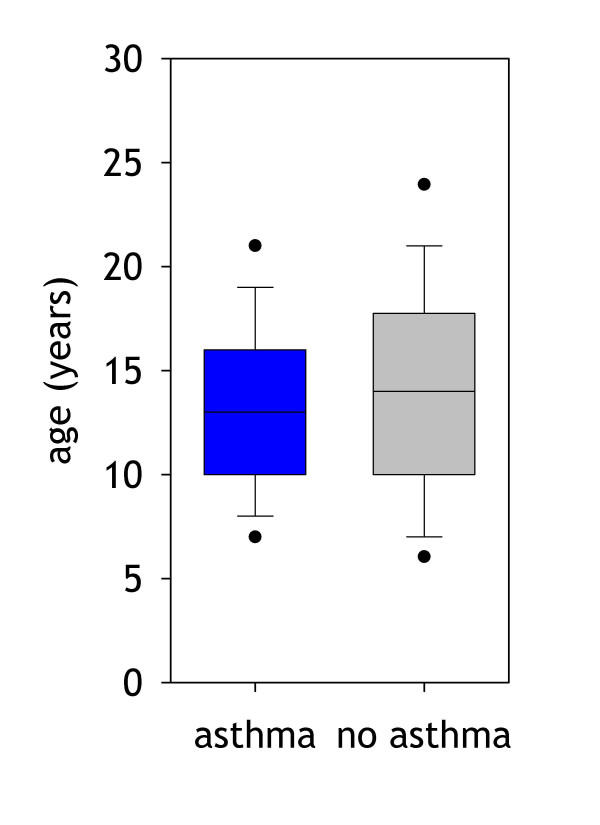
Age distribution in children with, blue box (left), and without asthma, grey box (right).

The TDT_DS _test as a global family test seems to capture the information both from affected and unaffected children, with four SNPs being at least marginally significantly associated at the 5% level. As unaffected children might be the younger children (that still have not developed a phenotype), the age distribution of affected and unaffected children was also compared (Fig. 3) but no difference was found. Affected and non affected children, however, show several other differences, all known as risk factors and symptoms for asthma: There are more boys in the affected group (57,6% vs. 36,1%), they are more often exposed to indoor environmental tobacco smoke (43.0% vs. 31.7%) and they are suffering more frequently from eczema (43,0% vs. 26,9%). Their average log(IgE) values are higher (7,7 kU/l vs. 5,8 kU/l), their forced 1 second capacity FEV1 is lower (2387 ml vs. 2625 ml) together with the forced vital capacity (2876 vs. 2998).

Bronchial hyper-reactivity (BHR), measured as quantitative trait locus (QTL) by using the slope of the dose-response curve in a standardized methacholine challenge protocol, also indicated an association with the same three SNPs identified in unaffected sibs. Further dichotomizing BHR by comparing the upper versus all other quartiles also yielded significant associations (hCV2880804, P = 0.005, rs1989969 P = 0.001, rs2853564 P = 0.001). The sum score of all specific IgE serum levels (RAST) was associated with markers rs1989969 (P = 0.021) and rs2853564 (P = 0.018), while total IgE was not found to be associated.

Table 2 summarizes the LD structure of all SNPs in the German families. LD was generally low, except for three SNPs on block "B". SNP rs2853563 probably does not interrupt block "B", as might be concluded from table 2, as this marker has a very low minor allele frequency (table 1). The overall low LD was very similar in the Swedish and Turkish subgroups of our families and are in line with recently published data [12-14]. The association seen for the two SNPs at the 3'-terminal region is probably influenced by the high LD between these two SNPs.

**Table 1:** Transmission disequilibrium of 13 VDR-SNPs in affected and unaffected children. P-values < 0.05 are indicated in bold.

						asthma				no asthma				
LD block *	dbSNP	allele	chr12 (bp)	geno-types	freq (%)	T	UT	T/UT	P	T	UT	T/UT	P	P TDT_DS_

A	hCV2880804	C	47954855	918	28	170	178	1,0	0,668	18	33	0,5	**0,049**	0,055
--	rs2228570	T	47977944	724	35	159	150	1,1	0,609	21	12	1,8	0,117	0,079
?	rs3819545	C	47981753	912	38	186	191	1,0	0,797	25	31	0,8	0,423	1,000
B	rs3782905	G	47982914	934	32	201	174	1,2	0,163	26	27	1,0	0,891	0,388
?	rs2853563	G	48248398	661	97	20	24	0,8	0,547	--	--	--	--	--
B	rs731236	C	48251417	923	37	202	206	1,0	0,843	25	29	0,9	0,586	0,767
B	rs1544410	A	48252495	896	37	190	186	1,0	0,837	23	26	0,9	0,668	1,000
B?	rs2239185	C	48257219	903	49	198	214	0,9	0,431	29	24	1,2	0,492	0,650
?	rs987849	C	48267336	825	45	164	169	1,0	0,784	28	18	1,6	0,140	0,173
B	rs1540339	A	48269986	916	36	189	187	1,0	0,575	25	27	0,9	0,782	0,706
?	rs2239186	C	48282070	929	19	116	145	0,8	0,073	20	18	1,1	0,746	0,189
C	rs1989969	T	48290670	922	43	204	198	1,0	0,765	39	20	2,0	**0,013**	**0,025**
C	rs2853564	C	48291147	920	44	196	201	1,0	0,802	38	20	1,9	**0,018**	**0,021**

*according to reference [15]

**Table 2:** D' matrix of 13 VDR-SNPs in German parents. D' values > = 0.69 are indicated in bold.

	rs2228570	rs3819545	rs3782905	rs2853563	rs731236	rs1544410	rs2239185	rs987849	rs1540339	rs2239186	rs1989969	rs2853564
hCV2880804	0,01	0,02	-0,04	0,05	0,01	0,01	-0,02	-0,01	0,05	0,02	-0,27	-0,27
Rs2228570		0,02	-0,03	0,01	0,06	0,02	-0,01	-0,03	-0,03	-0,10	0,12	0,13
Rs3819545			-0,53	0,08	-0,41	-0,41	0,26	0,25	**0,85**	0,58	-0,02	-0,02
Rs3782905				-0,08	0,53	0,55	-0,44	-0,45	-0,45	-0,33	0,05	0,05
Rs2853563					-0,14	-0,16	0,20	-0,10	0,19	-0,01	-0,07	-0,08
Rs731236						**0,98**	**-0,76**	**-0,69**	-0,46	-0,32	0,07	0,05
Rs1544410							**-0,76**	**-0,69**	-0,46	-0,33	0,06	0,05
Rs2239185								**0,85**	0,28	0,33	-0,06	-0,05
Rs987849									0,22	0,40	-0,02	-0,01
Rs1540339										0,58	-0,07	-0,07
Rs2239186											-0,11	-0,11
Rs1989969												**0,98**

Finally, a 4-locus haplotype was constructed of those SNPs associated in the TDT_DS_. Again, no significant transmission distortion was found in affected children, while one haplotype showed a 5.1-fold over-transmission in unaffected children (P = 0.009).

## Discussion

This study addresses a previously described association of VDR SNPs with asthma and related phenotypic traits. Although a preferential transmission of vitamin D receptor variants to children with asthma could not be confirmed, it raises the possibility of a protective effect in unaffected children. Although the effect is rather moderate, several SNPs support this association. If any, the association is probably more related to RNA turnover than to a structural modification of the receptor as there was no association with LD block "B" that codes for the translated exons. Gene expression can be varied over a 100-fold range by subtle modifications of the 3'-terminal sequence [15], which will requires further research on the function of these allelic variants in target tissues.

This notion is partially in contrast with a previous study of 7 VDR SNPs in the CAMP (Childhood Asthma Management Program) study of 582 nuclear families where SNP rs7975232 (akin ApaI) in intron 8 showed a highly significant effect [12]. A confirmation study by the same group in a case-control sample of the Nurses' Health Study NHS [12] also associated asthma with rs3782905 (intron 2, P = 0.02), rs2239185 (intron 3, P = 0.02) and rs731236 (Ile352Ile, P = 0.03). A study of 223 independent Canadian families reported six out of twelve SNPs to be associated with asthma (rs3782905, rs1540339, rs2239182, rs2239185, BsmI, ApaI, TaqI), most of these on block "B". As none of these SNPs was giving rise to an amino acid change the authors speculated about an intronic regulatory SNP or one or more functional variants at the 3' end of the VDR locus.

It is unlikely that increased or decreased vitamin D sensitivity is simply mediated by a genetic variation in the VDR. Vitamin D requires several enzymatic steps to be activated, transported and degraded; receptor signalling requires several co-factors and all of these may contribute additive or multiplicative effects on vitamin D sensitivity. The co-activator retinoic acid receptor RXR may itself affect Th1 and Th2 development [16]. Other transcription factors involved are SRC/p160, CBP and p300 [17]. The DRIP complex attaches to the VDR/RXR complex and binds to vitamin D responsive elements (VDRE) through histone acetyltransferase activity. Importantly, some of the vitamin D regulated genes are also located in allergy linkage regions. The renal 1-α-hydroxylase (12q13) and the 24-hydroxylase (20q13), as well as RXR (6p21), are all positional candidates. RXR has already been tagged by a SNP in a previous study [18] and also 3 SNPs in CYP24A1 (rs751089, rs2296241 and rs2248137) were significantly associated with asthma (*unpublished own observation*). CYP24A1 is particular interesting as it is the major enzyme of the degradation pathway that showed a 97-fold increase after vitamin D treatment of rats [19] or 12-fold increase in a human colon cancer cell line [20].

The number of genes regulated by vitamin D has been recently extended beyond those genes with known VDRE promoter motifs (calbindin, PTH, PTHRP, ITGB3, OC, GH, osteopontin, osteocalcin, c-fos, IL-2Rβ, NFκB, sCD23) to another 150 up- and down-regulated regulated genes [19-23]. The interaction of genetic variants in the VDR and other positional, as well as functional, candidate genes is therefore a current research topic. So far, only two gene associations have been published: Vitamin D binding protein (GC*2 and GC*1F) was associated with an increased risk for COPD [24,25] and osteopontin (OPN C8090T and T9250C) with increased total IgE in asthmatic patients [26].

In addition to its biological context, this study also has some implications on the statistical analysis as the rather low number of unaffected sibs seemed to contribute to a few positive associations. Despite enormous efforts to map complex genetic diseases, SNP association studies are often lacking power [27]. Linkage studies in affected sib pairs have been preferably used to map complex diseases to chromosomal regions [9], while "no substantial study of normal sib-pairs has been undertaken, making this family of surveys one of the largest undertaken in the absence of controls" [28] although unselected affected sib pairs tend to share more than half of their alleles [29]. This omission is even more remarkable as discordant sib pairs (DSP) have been shown to be a more powerful alternative [30]. Risch proposed to test DSP in the top ten and bottom ten percent distribution of quantitative traits, as pairs with intermediate values (between the 30^th ^and 70^th ^percentiles) did not provide much information for linkage analysis [31]. Although the DSP concept was appealing from a theoretical standpoint, it turned out that there are disadvantages for practical reasons. It requires a large amount of individuals to be screened, which might be the reason that the DSP approach has not received the expected attention except for a few studies (for a summary see [31]).

Transmission disequilibrium testing of unaffected child – parent trios originating from families with another two affected offsprings, may be a powerful alternative. As there is a strong ascertainment bias of these families toward a genetic risk, as well as a disease causing environmental factor, being unaffected is an extreme phenotype ("being sane in an insane world"). The transmission to unaffected children can be seen as an independent cross-match to the transmission to affected children. The high power of testing unaffected sibs has already been predicted on theoretical grounds [32]. The number of DSPs required to achieve 80% power (with a difference in the allele frequency of 15% and λ_s _of 3.2) has been estimated to be approx. 250 [33]. A sample size of 1,500 families was estimated by including two affected and one unaffected children [34]. In this study already 50 DSPs were sufficient to show a significant distortion in the allele transmission. Non-paternity as a reason for discordant traits [35] is unlikely as nearly all families were included in a previous genome-wide scan.

## Conclusions

The transmission disequilibrium in unaffected sibs in otherwise multiple-affected families seems to be a powerful test. A preferential transmission of vitamin D receptor variants to children with asthma could not be confirmed but raises the possibility of a protective effect in unaffected children.

## Methods

### Study population

The German asthma sib pair families were collected in 26 paediatric centres in Germany and Sweden for an initial genome-wide linkage scan. In these families at least two children were required with confirmed doctor-diagnosed asthma, while prematurity or low birth weight of the children were excluded, along with any other severe pulmonary disease. All affected children should have after their 3^rd ^birthday a history of at least three years of recurrent wheezing and should not have any other airway disease diagnosed. Unaffected siblings were also sampled if they were at least 6 years old, eligible for pulmonary function testing and did not have doctor-diagnosed asthma.

On the first home visit a complete pedigree of the family was drawn and information collected in a questionnaire. Participants were examined for several associated phenotypes. Pulmonary function tests were performed by forced flow volume tests and bronchial challenge was done by methacholine.

Briefly, pulmonary function tests were performed by forced expiration in a sitting position using a nose-clip. Forced flow volume tests were performed until three reproducible loops were achieved. Of these the trial with the maximum sum of FVC and FEV1.0 was used for the analysis. Bronchial challenge with methacholine was done with increasing doses of 0, 0.156, 0.312, 0.625, 1.25, 2.5, 5, 10, 25 mg/ml during 5 consecutive breaths with 14 mg delivered from a de Vilbiss 646 nebulizer chamber by using a breath-triggered pump ZAN 200 (Zan, Oberthulpa, Germany). The provocation was stopped either with the occurrence of symptoms or a fall of 20% from the baseline FEV1.0 and the slope of the dose-response curve calculated.

Total IgE was determined with an ELISA (Pharmacia Diagnostics, Uppsala, Sweden). The allergens tested were birch (betula verruscose) ALK SQ108, hazel (corylus avellana) ALK SQ113, the herbs ribworth (plantago lanceolata) ALK N342 and mugwort ALK SQ312, mixed grass ALK SQ299, dust mite dermatophagoides farinae ALK SQ 504, dermatophagoides pteronyssimus ALK SQ 503, cat dander ALK SQ555 and dog dander ALK SQ 553, and fungi (aspergillus fumigatus ALK N405 and alternaria alternata ALK N402), which were bought in one batch and stored at +5°C until analysis.

The original family collection [36] has been expanded since 1994 to a larger sample, of which 218 families were available in 2002 for a complete genome scan. The consecutive families were tested with the same protocol as described earlier [36] except that the time-consuming methacholine challenge protocol was omitted. Excluded from the final sample were individuals with incomplete data, missing DNA samples, identical twins and all probands with more than 2 non-segregating out of 408 microsatellite markers. Another 6 families could be included in this analysis, resulting in a final sample of 224 families. Each study participant, including all children, signed a consent form. All study methods were approved in 1995 by the ethics commission of "Nordrhein-Westfalen" and again in 2001 by "Bayerische Landesärztekammer München".

### DNA preparation and genotyping

DNA was isolated from peripheral white blood cells using Qiamp (Qiagen, Germany) or Puregene isolation kits (Gentra Systems, Minneapolis, MN, USA). From the VDR we selected 16 SNPs where 13 could finally be analyzed. They had to be polymorphic, complete, received a high calling score, passed paternity checks and were in Hardy-Weinberg equilibrium. The following three markers have been excluded from the analysis: rs2239179 (contaminant in mass spectrum peak), rs2853559 (Hardy-Weinberg disequilibrium, paternity errors) and rs797523 (typing error in primer sequence).

Genotyping was performed using MALDI-TOF mass spectrometry of allele-specific primer extension products (Table 3) generated from amplified DNA sequences (MassARRAY, SEQUENOM Inc., San Diego, CA, USA). Primers were obtained from Metabion GmbH (Planegg-Martinsried, Germany).

**Table 3:** Primer used for genotyping

SNP	left primer	right primer	extension primer	stop mix
hCV2880804	ACGTTGGATG-CTGGGATACTTCTGAGAGTG	ACGTTGGATG-TTTTCCCTGGAAAGTTTGGG	CCATTGTCCTGGTATAACCA	ACG
rs2228570	ACGTTGGATG-TCAAAGTCTCCAGGGTCAGG	ACGTTGGATG-AGACCTCACAGAAGAGCACC	CCCTGCTCCTTCAGGGA	ACG
rs3819545	ACGTTGGATG-AATGGTGGTTACTGCAGCTC	ACGTTGGATG-GAAACCAGTCCTCTGTCATG	TAGGTTCGGTCTTTGGCT	ACG
rs3782905	ACGTTGGATG-GGGTCTCAAATTCTTAATGAG	ACGTTGGATG-AAACTAGCAGAAAGAGGCAG	GTGGGAGGGAGTGCTGA	ACT
rs2853564	ACGTTGGATG-CTGCATTGCTCCTGACTTAG	ACGTTGGATG-AGGTAGCTTAGCTCTGAGTC	TTTCTGCAACCCTAAGCC	ACT
rs731236	ACGTTGGATG-TGTGCCTTCTTCTCTATCCC	ACGTTGGATG-TGTACGTCTGCAGTGTGTTG	CGGTCCTGGATGGCCTC	ACT
rs1544410	ACGTTGGATG-TAGATAAGCAGGGTTCCTGG	ACGTTGGATG-AATGTTGAGCCCAGTTCACG	AGCCTGAGTATTGGGAATG	ACG
rs2239185	ACGTTGGATG-CAATTCCAGTCACATCTCGG	ACGTTGGATG-CCTGTGTGACATTTACACCC	CCCTCCTCTGTCTTCAC	ACT
rs987849	ACGTTGGATG-GAATAGTGCCTTATAGATAG	ACGTTGGATG-AGCTAGAAGTTCTGGTGATC	GAAATATTCGTAATGCTGGAT	ACT
rs1540339	ACGTTGGATG-TCACACACATTCTCAGTGGG	ACGTTGGATG-TTTGCAGAGGCTGTCTTCTC	GTTGGTGCCCACCCTAA	ACG
rs2239186	ACGTTGGATG-GTCCACAGTGACTATAGACC	ACGTTGGATG-AAGAAGGAGAAGCAGGCATC	CAGGGGTGGAAGAAGAGGAG	ACT
rs1989969	ACGTTGGATG-TGTATGCAGAGCTTAGCAGG	ACGTTGGATG-TTTCAGAGGTCAGAGGTGAC	GTCAGAGGTGACATCCAG	ACT
rs2853564	ACGTTGGATG-CTGCATTGCTCCTGACTTAG	ACGTTGGATG-AGGTAGCTTAGCTCTGAGTC	TTTCTGCAACCCTAAGCC	ACT

### Data handling and statistical analysis

Clinical data and genotypes were transferred to a SQL 2000 database by using Cold Fusion 4.0 scripts. Statistical analyses were performed using R 1.8.1 [37] by accessing the database with the RODBC module. For each SNP, the distribution of genotypes in pseudo-controls created by combining the parental alleles not transmitted to asthma children was tested by a χ^2^-test as well as the transmission to their unaffected sibs. An extension of the classical TDT [34] was also implemented that incorporates effectively both affected and unaffected children (TDT_DS_). The standardized linkage disequilibrium coefficient D' and the correlation coefficient R^2 ^were calculated for each pair of SNPs in parents by using the R package "genetics". All analyses were cross-checked by using SIBPAIR software. Haplotypes were estimated using TDTPHASED in the UNPHASED package [38] and transmission to affected and unaffected children tested separately. For phase-certain haplotypes a conditional logistic regression model was used, corresponding to the probability of the offspring conditional upon the parents. When phase was uncertain, unconditional logistic regression on the full likelihood of parents and offspring was used instead (see [39] for the formulations of these likelihoods). Transmitted haplotypes were compared to all untransmitted haplotypes, equivalent to the haplotype-based haplotype relative risk, while an EM algorithm was used to obtain maximum-likelihood estimates of case and control parental haplotype frequencies under both null and alternative hypotheses.

## Abbreviations

VDR vitamin D receptor

VDRE vitamin D responsive element

SNP single nucleotide polymorphism

MALDI-TOF matrix assisted laser desorption ionisation – time of flight

DSP discordant sib pairs

TDT transmission disequilibrium

LD linkage disequilibrium

QTL quantitative trait locus

BHR bronchial hyperreactivity

FEV1 forced volume during the 1st second

## Author's contribution

The author developed the idea presented in this paper, initiated the study, applied for funding, developed protocols, participated in the clinical survey, planned the laboratory analysis, did the statistical analysis, and drafted the report. This manuscript contains no patient identifiable information.
